# Supplementary material for: The NUCKS1-SKP2-p21/p27 axis controls S phase entry
Source: Nat Commun. 2021 Nov 29;12:6959. doi: 10.1038/s41467-021-27124-8 (PMC8630071; doi:10.1038/s41467-021-27124-8)
Supplement: Supplementary file 1 — Supplementary Information [file 41467_2021_27124_MOESM1_ESM.pptx]

## Slide 1
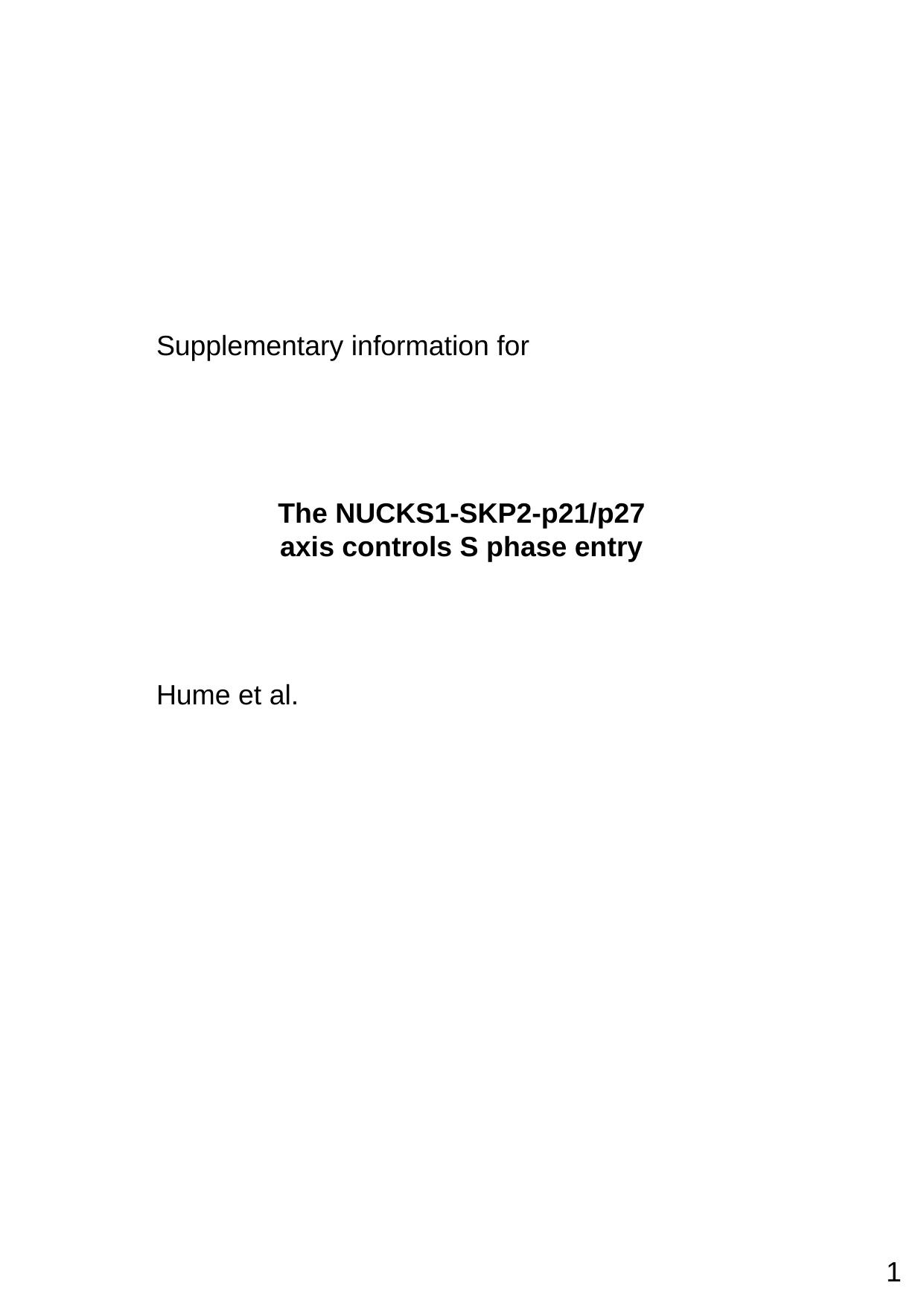

Supplementary information for
The NUCKS1-SKP2-p21/p27 axis controls S phase entry
Hume et al.
1

## Slide 2
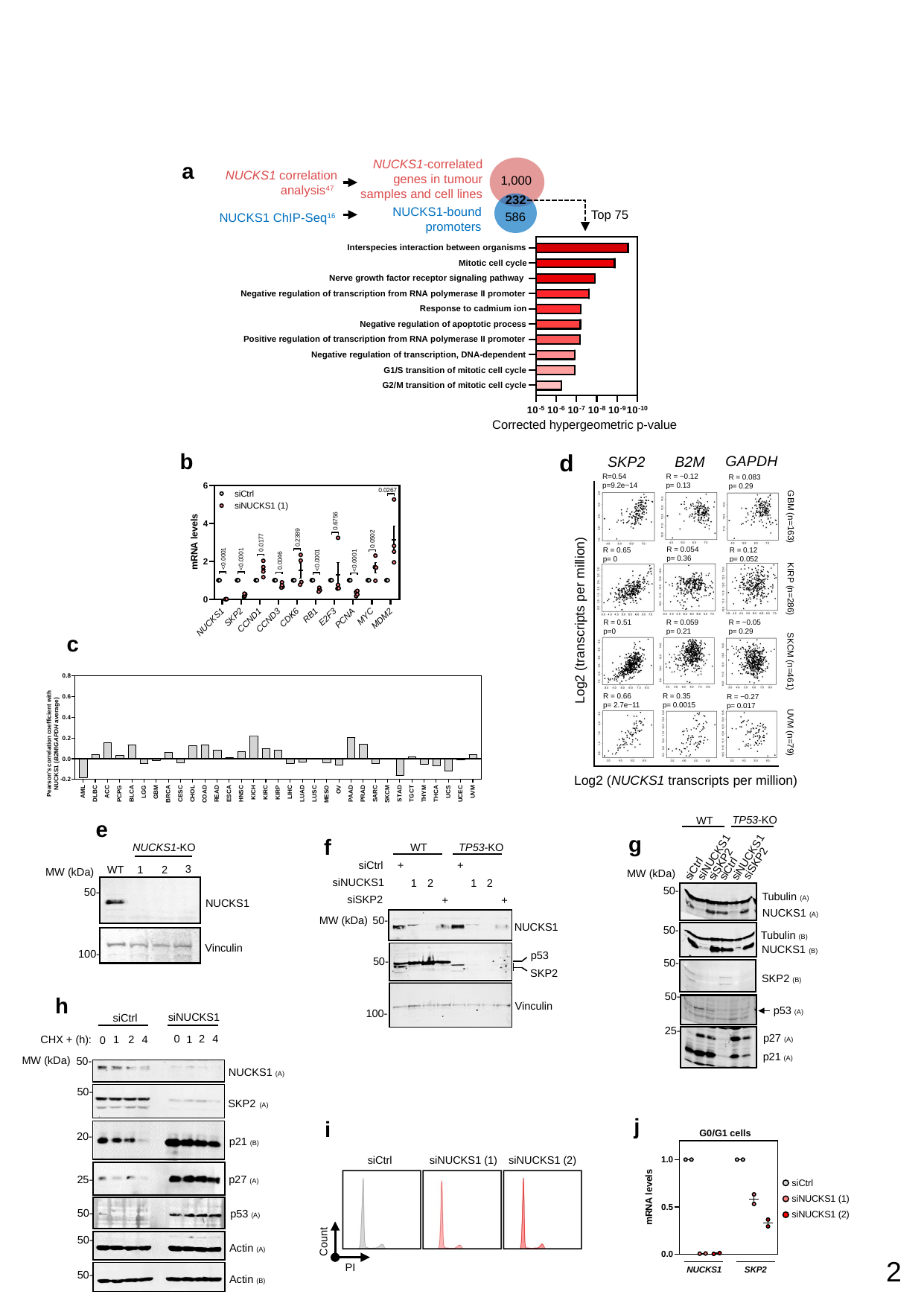

NUCKS1-correlated genes in tumour samples and cell lines
NUCKS1 correlation analysis47
1,000
232
NUCKS1-bound promoters
Top 75
586
NUCKS1 ChIP-Seq16
a
b
d
GAPDH
B2M
SKP2
R=0.54
p=9.2e−14
R = −0.12
p= 0.13
R = 0.083
p= 0.29
GBM (n=163)
R = 0.054
p= 0.36
R = 0.65
p= 0
R = 0.12
p= 0.052
Log2 (transcripts per million)
KIRP (n=286)
R = 0.059
p= 0.21
R = 0.51
p=0
R = −0.05
p= 0.29
c
SKCM (n=461)
R = −0.27
p= 0.017
R = 0.66
p= 2.7e−11
R = 0.35
p= 0.0015
UVM (n=79)
Log2 (NUCKS1 transcripts per million)
TP53-KO
WT
e
g
f
WT
NUCKS1-KO
3
2
1
WT
50-
NUCKS1
Vinculin
100-
TP53-KO
siNUCKS1
siNUCKS1
siSKP2
siSKP2
siCtrl
+
+
siCtrl
siCtrl
MW (kDa)
MW (kDa)
siNUCKS1
1
2
1
2
50-
Tubulin (A)
siSKP2
+
+
NUCKS1 (A)
MW (kDa)
50-
NUCKS1
50-
Tubulin (B)
NUCKS1 (B)
p53
SKP2
50-
50-
SKP2 (B)
50-
h
Vinculin
p53 (A)
100-
siNUCKS1
siCtrl
25-
p27 (A)
0
2
4
1
4
2
CHX + (h):
1
0
p21 (A)
MW (kDa)
50-
NUCKS1 (A)
50-
SKP2 (A)
j
i
20-
p21 (B)
siNUCKS1 (2)
siCtrl
siNUCKS1 (1)
Count
PI
p27 (A)
25-
50-
p53 (A)
50-
Actin (A)
2
50-
Actin (B)

## Slide 3
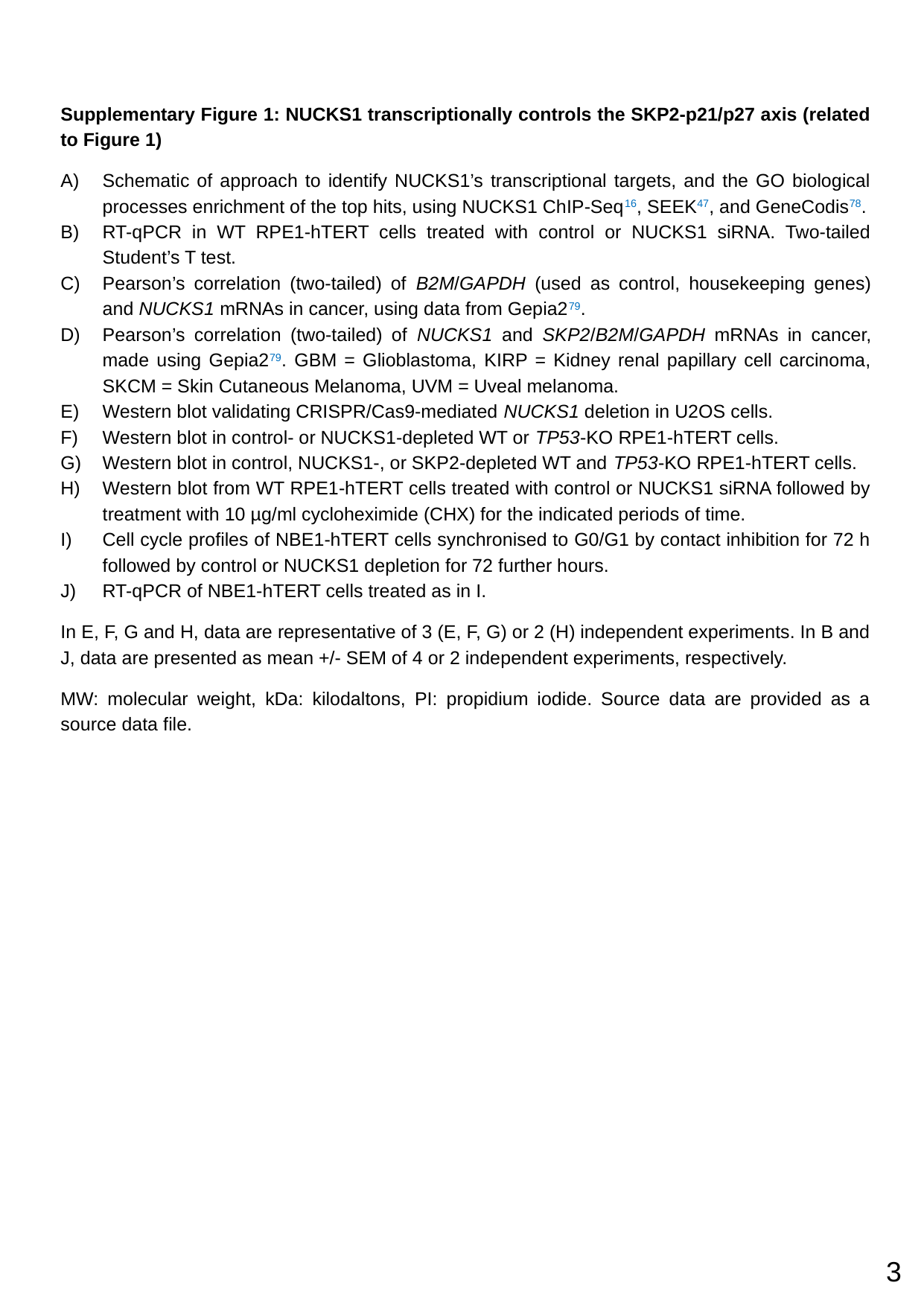

Supplementary Figure 1: NUCKS1 transcriptionally controls the SKP2-p21/p27 axis (related to Figure 1)
Schematic of approach to identify NUCKS1’s transcriptional targets, and the GO biological processes enrichment of the top hits, using NUCKS1 ChIP-Seq16, SEEK47, and GeneCodis78.
RT-qPCR in WT RPE1-hTERT cells treated with control or NUCKS1 siRNA. Two-tailed Student’s T test.
Pearson’s correlation (two-tailed) of B2M/GAPDH (used as control, housekeeping genes) and NUCKS1 mRNAs in cancer, using data from Gepia279.
Pearson’s correlation (two-tailed) of NUCKS1 and SKP2/B2M/GAPDH mRNAs in cancer, made using Gepia279. GBM = Glioblastoma, KIRP = Kidney renal papillary cell carcinoma, SKCM = Skin Cutaneous Melanoma, UVM = Uveal melanoma.
Western blot validating CRISPR/Cas9-mediated NUCKS1 deletion in U2OS cells.
Western blot in control- or NUCKS1-depleted WT or TP53-KO RPE1-hTERT cells.
Western blot in control, NUCKS1-, or SKP2-depleted WT and TP53-KO RPE1-hTERT cells.
Western blot from WT RPE1-hTERT cells treated with control or NUCKS1 siRNA followed by treatment with 10 µg/ml cycloheximide (CHX) for the indicated periods of time.
Cell cycle profiles of NBE1-hTERT cells synchronised to G0/G1 by contact inhibition for 72 h followed by control or NUCKS1 depletion for 72 further hours.
RT-qPCR of NBE1-hTERT cells treated as in I.
In E, F, G and H, data are representative of 3 (E, F, G) or 2 (H) independent experiments. In B and J, data are presented as mean +/- SEM of 4 or 2 independent experiments, respectively.
MW: molecular weight, kDa: kilodaltons, PI: propidium iodide. Source data are provided as a source data file.
3

## Slide 4
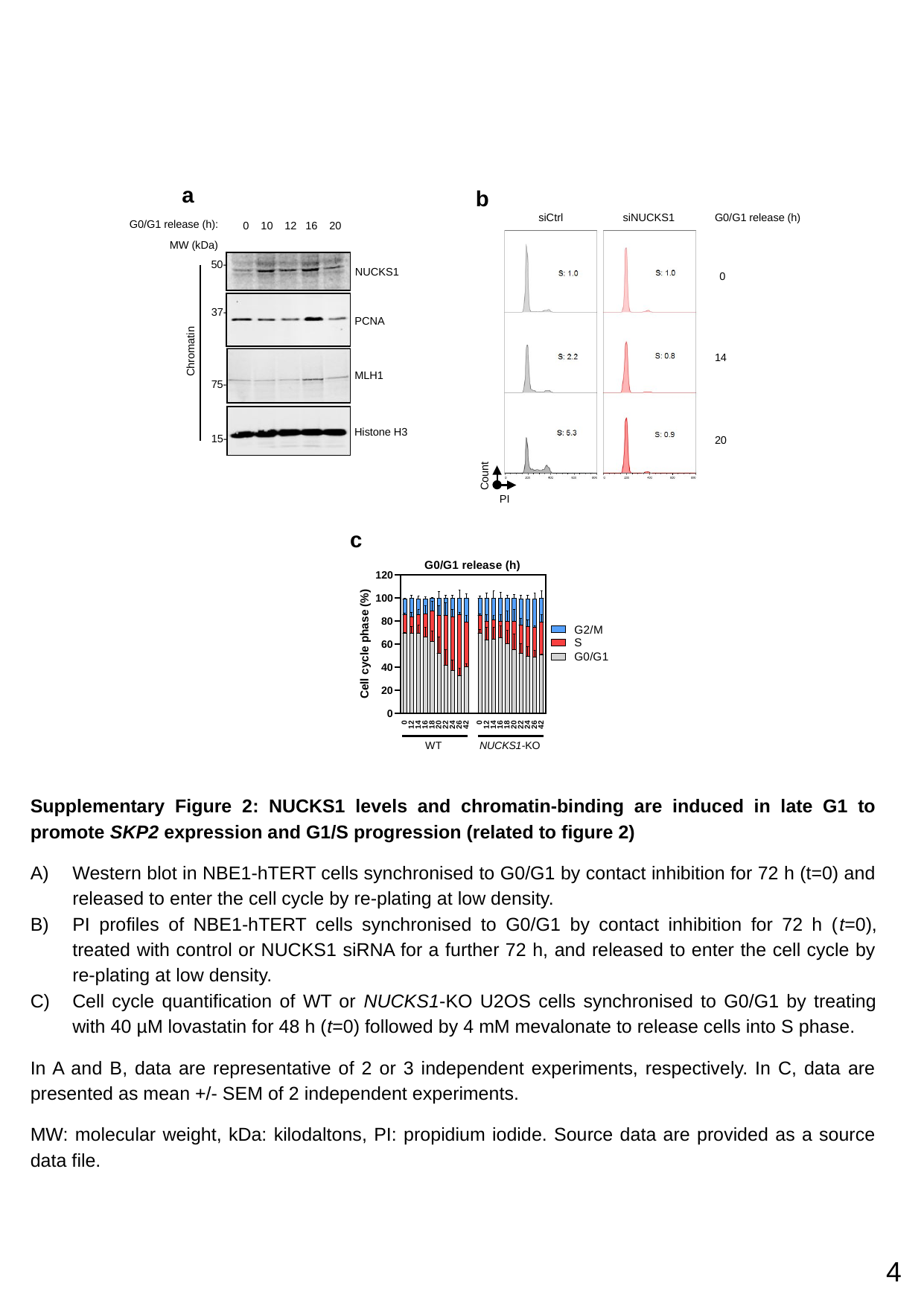

a
b
siNUCKS1
siCtrl
G0/G1 release (h)
G0/G1 release (h):
0 10 12 16 20
MW (kDa)
50-
NUCKS1
0
37-
PCNA
Chromatin
14
MLH1
75-
Histone H3
15-
20
Count
PI
c
Supplementary Figure 2: NUCKS1 levels and chromatin-binding are induced in late G1 to promote SKP2 expression and G1/S progression (related to figure 2)
Western blot in NBE1-hTERT cells synchronised to G0/G1 by contact inhibition for 72 h (t=0) and released to enter the cell cycle by re-plating at low density.
PI profiles of NBE1-hTERT cells synchronised to G0/G1 by contact inhibition for 72 h (t=0), treated with control or NUCKS1 siRNA for a further 72 h, and released to enter the cell cycle by re-plating at low density.
Cell cycle quantification of WT or NUCKS1-KO U2OS cells synchronised to G0/G1 by treating with 40 µM lovastatin for 48 h (t=0) followed by 4 mM mevalonate to release cells into S phase.
In A and B, data are representative of 2 or 3 independent experiments, respectively. In C, data are presented as mean +/- SEM of 2 independent experiments.
MW: molecular weight, kDa: kilodaltons, PI: propidium iodide. Source data are provided as a source data file.
4

## Slide 5
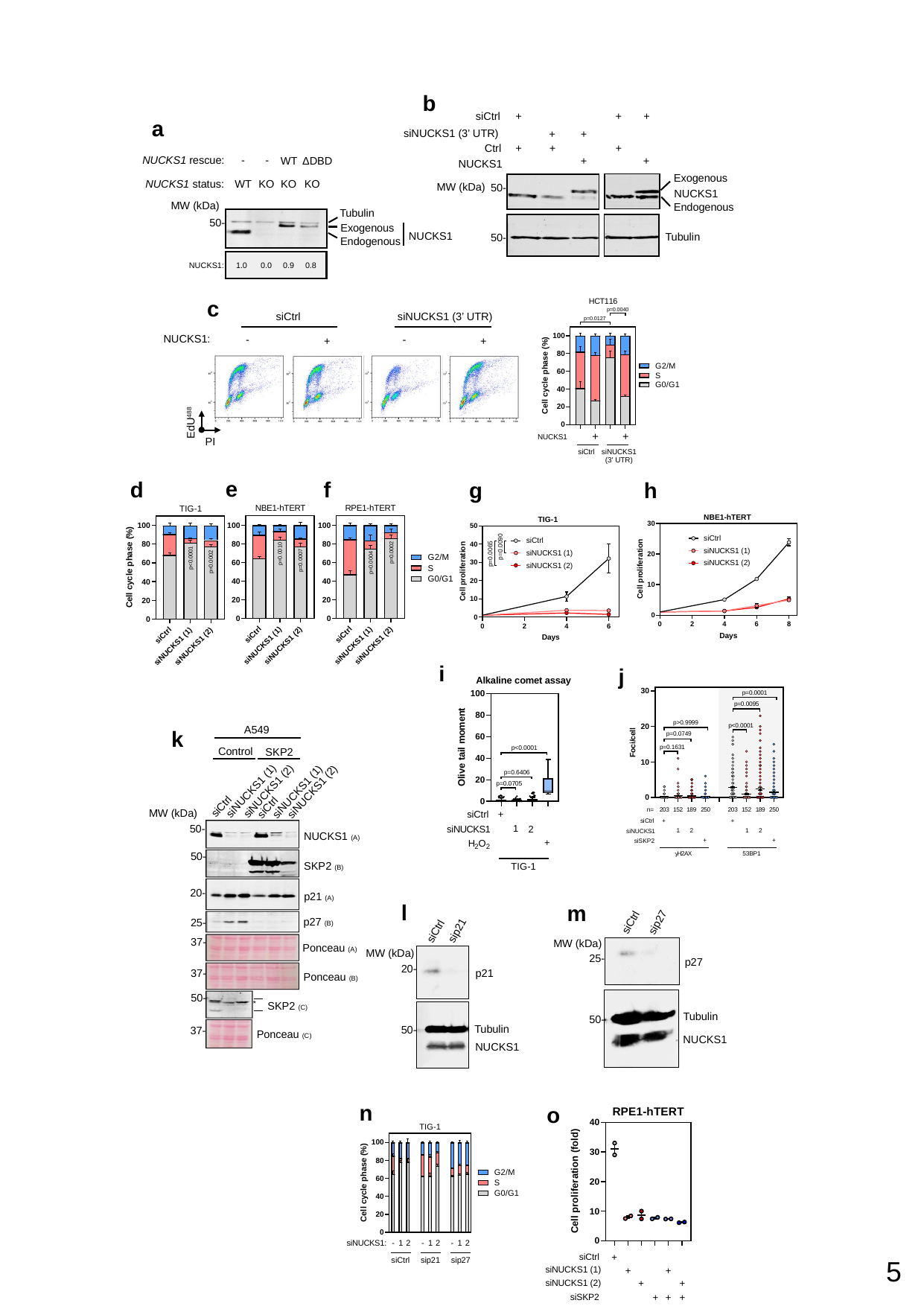

b
siCtrl
+
+
+
a
siNUCKS1 (3’ UTR)
+
+
Ctrl
+
+
+
-
-
NUCKS1 rescue:
+
+
ΔDBD
WT
NUCKS1
Exogenous
KO
KO
WT
KO
NUCKS1 status:
MW (kDa)
50-
NUCKS1
MW (kDa)
Endogenous
Tubulin
50-
Exogenous
NUCKS1
Tubulin
50-
Endogenous
0.9
0.8
1.0
0.0
NUCKS1:
c
siNUCKS1 (3’ UTR)
siCtrl
NUCKS1:
-
-
+
+
EdU488
PI
e
d
f
h
g
i
j
A549
k
Control
SKP2
siNUCKS1 (2)
siNUCKS1 (1)
siNUCKS1 (2)
siNUCKS1 (1)
siCtrl
siCtrl
MW (kDa)
50-
NUCKS1 (A)
50-
SKP2 (B)
20-
p21 (A)
l
m
siCtrl
sip27
p27 (B)
siCtrl
25-
sip21
37-
MW (kDa)
Ponceau (A)
MW (kDa)
25-
p27
20-
p21
37-
Ponceau (B)
50-
*
SKP2 (C)
Tubulin
50-
Tubulin
50-
37-
Ponceau (C)
NUCKS1
NUCKS1
n
o
5

## Slide 6
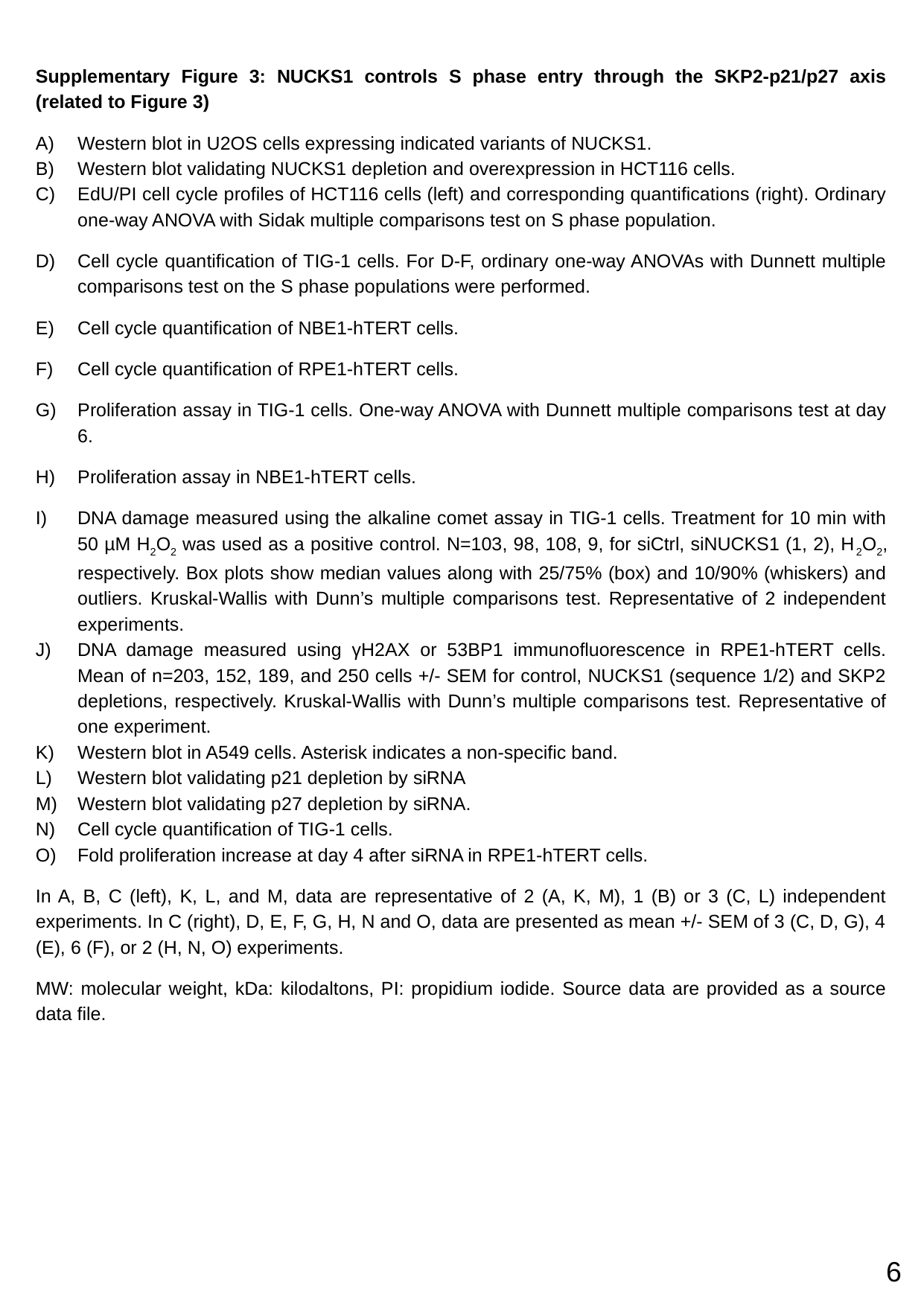

Supplementary Figure 3: NUCKS1 controls S phase entry through the SKP2-p21/p27 axis (related to Figure 3)
Western blot in U2OS cells expressing indicated variants of NUCKS1.
Western blot validating NUCKS1 depletion and overexpression in HCT116 cells.
EdU/PI cell cycle profiles of HCT116 cells (left) and corresponding quantifications (right). Ordinary one-way ANOVA with Sidak multiple comparisons test on S phase population.
Cell cycle quantification of TIG-1 cells. For D-F, ordinary one-way ANOVAs with Dunnett multiple comparisons test on the S phase populations were performed.
Cell cycle quantification of NBE1-hTERT cells.
Cell cycle quantification of RPE1-hTERT cells.
Proliferation assay in TIG-1 cells. One-way ANOVA with Dunnett multiple comparisons test at day 6.
Proliferation assay in NBE1-hTERT cells.
DNA damage measured using the alkaline comet assay in TIG-1 cells. Treatment for 10 min with 50 µM H2O2 was used as a positive control. N=103, 98, 108, 9, for siCtrl, siNUCKS1 (1, 2), H2O2, respectively. Box plots show median values along with 25/75% (box) and 10/90% (whiskers) and outliers. Kruskal-Wallis with Dunn’s multiple comparisons test. Representative of 2 independent experiments.
DNA damage measured using γH2AX or 53BP1 immunofluorescence in RPE1-hTERT cells. Mean of n=203, 152, 189, and 250 cells +/- SEM for control, NUCKS1 (sequence 1/2) and SKP2 depletions, respectively. Kruskal-Wallis with Dunn’s multiple comparisons test. Representative of one experiment.
Western blot in A549 cells. Asterisk indicates a non-specific band.
Western blot validating p21 depletion by siRNA
Western blot validating p27 depletion by siRNA.
Cell cycle quantification of TIG-1 cells.
Fold proliferation increase at day 4 after siRNA in RPE1-hTERT cells.
In A, B, C (left), K, L, and M, data are representative of 2 (A, K, M), 1 (B) or 3 (C, L) independent experiments. In C (right), D, E, F, G, H, N and O, data are presented as mean +/- SEM of 3 (C, D, G), 4 (E), 6 (F), or 2 (H, N, O) experiments.
MW: molecular weight, kDa: kilodaltons, PI: propidium iodide. Source data are provided as a source data file.
6

## Slide 7
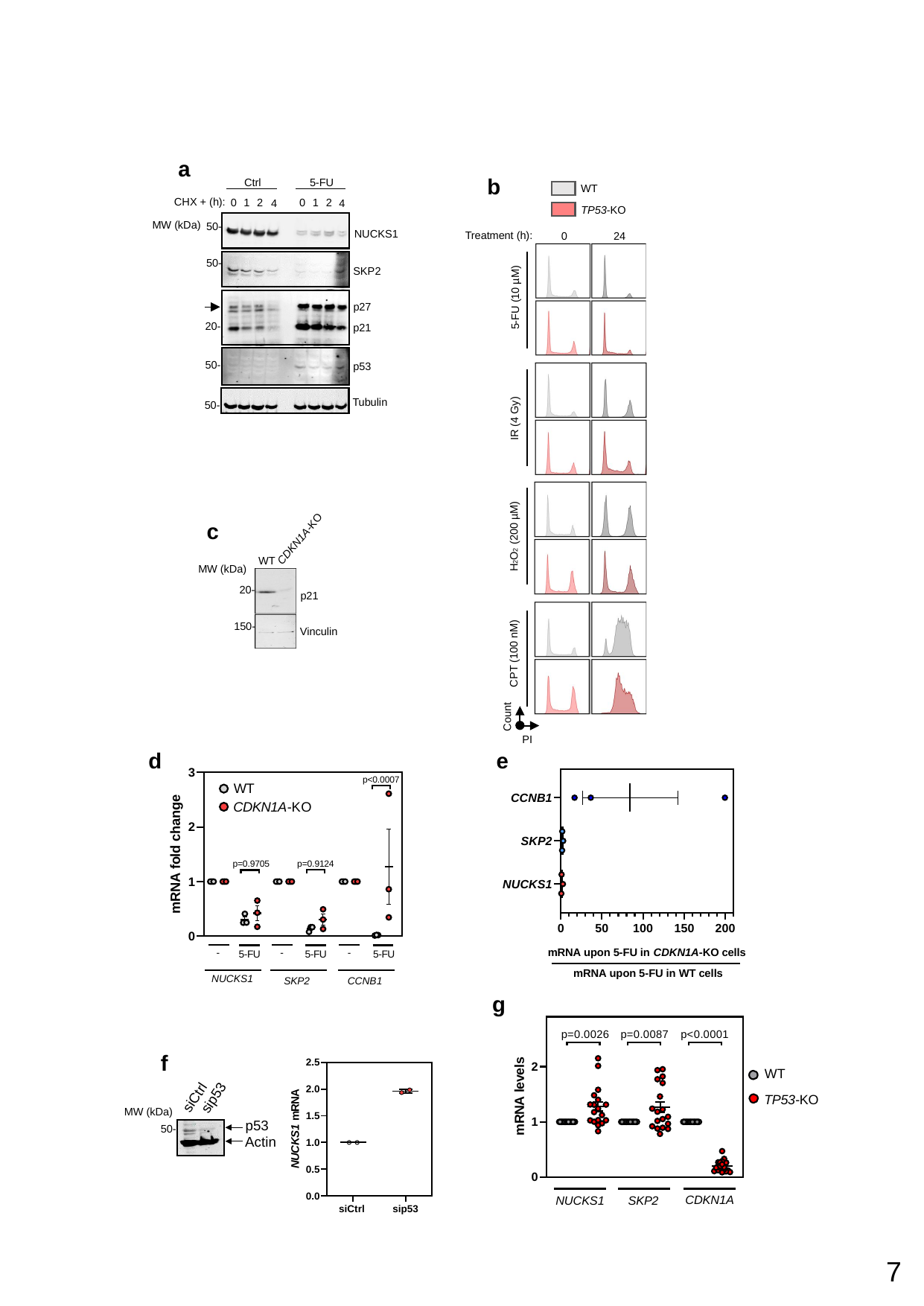

a
b
Ctrl
5-FU
WT
TP53-KO
CHX + (h):
0
2
1
0
2
1
4
4
MW (kDa)
50-
NUCKS1
Treatment (h):
24
0
50-
SKP2
5-FU (10 µM)
p27
20-
p21
50-
p53
Tubulin
50-
IR (4 Gy)
CDKN1A-KO
WT
20-
p21
150-
Vinculin
c
H2O2 (200 µM)
MW (kDa)
CPT (100 nM)
Count
PI
d
e
g
f
siCtrl
sip53
MW (kDa)
p53
50-
Actin
7

## Slide 8
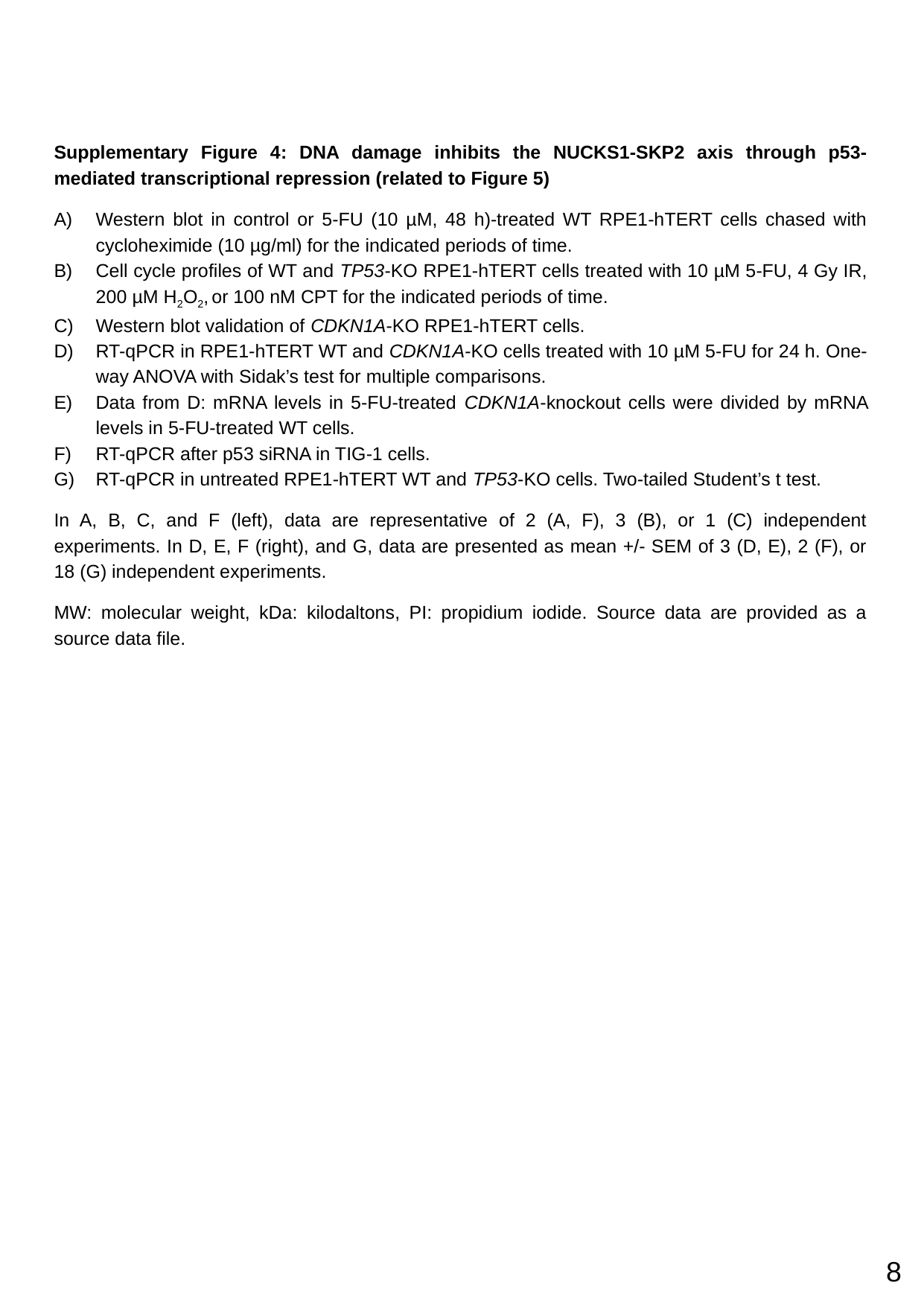

Supplementary Figure 4: DNA damage inhibits the NUCKS1-SKP2 axis through p53-mediated transcriptional repression (related to Figure 5)
Western blot in control or 5-FU (10 µM, 48 h)-treated WT RPE1-hTERT cells chased with cycloheximide (10 µg/ml) for the indicated periods of time.
Cell cycle profiles of WT and TP53-KO RPE1-hTERT cells treated with 10 µM 5-FU, 4 Gy IR, 200 µM H2O2, or 100 nM CPT for the indicated periods of time.
Western blot validation of CDKN1A-KO RPE1-hTERT cells.
RT-qPCR in RPE1-hTERT WT and CDKN1A-KO cells treated with 10 µM 5-FU for 24 h. One-way ANOVA with Sidak’s test for multiple comparisons.
Data from D: mRNA levels in 5-FU-treated CDKN1A-knockout cells were divided by mRNA levels in 5-FU-treated WT cells.
RT-qPCR after p53 siRNA in TIG-1 cells.
RT-qPCR in untreated RPE1-hTERT WT and TP53-KO cells. Two-tailed Student’s t test.
In A, B, C, and F (left), data are representative of 2 (A, F), 3 (B), or 1 (C) independent experiments. In D, E, F (right), and G, data are presented as mean +/- SEM of 3 (D, E), 2 (F), or 18 (G) independent experiments.
MW: molecular weight, kDa: kilodaltons, PI: propidium iodide. Source data are provided as a source data file.
8

## Slide 9
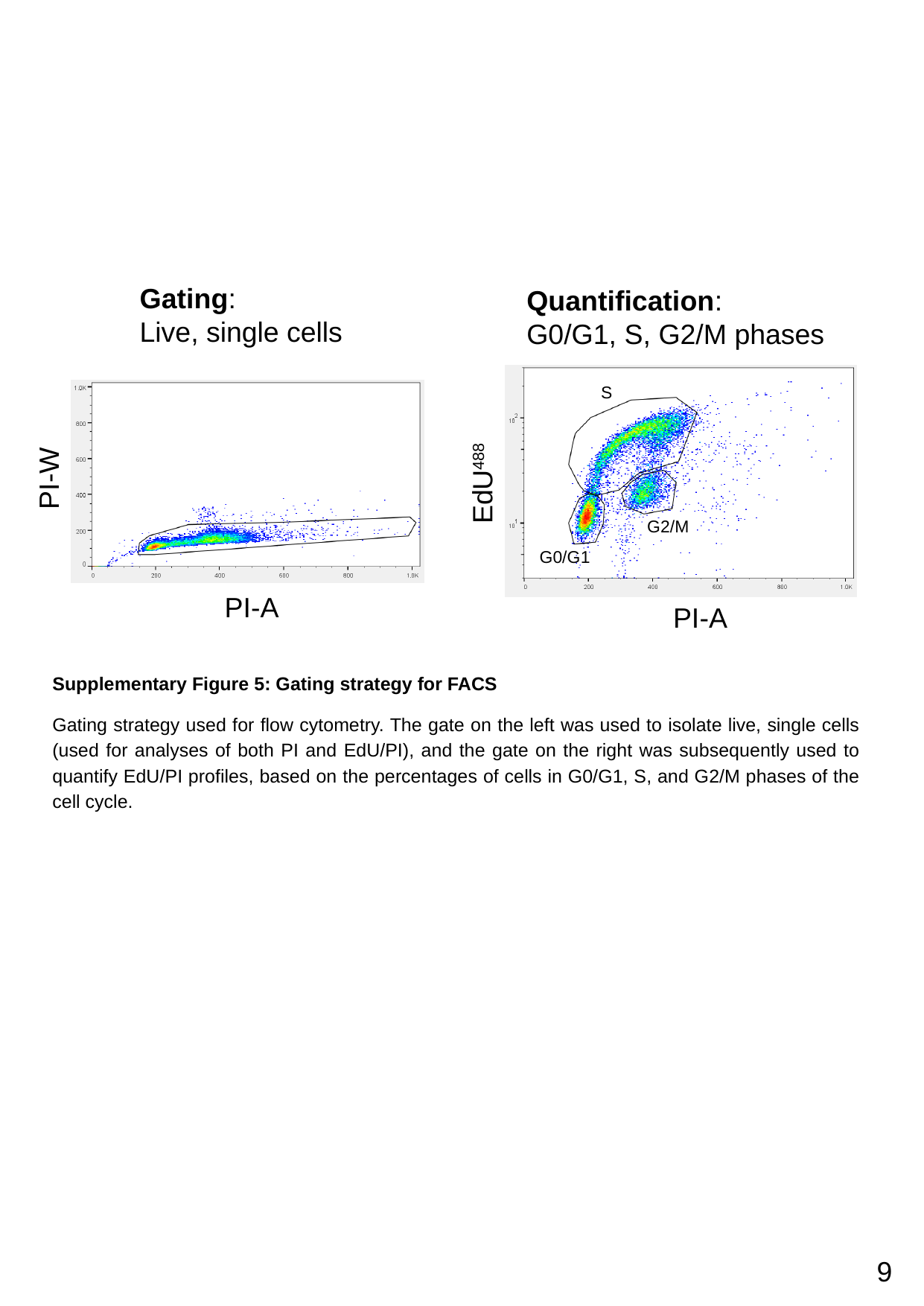

Gating:
Live, single cells
Quantification:
G0/G1, S, G2/M phases
S
PI-W
EdU488
G2/M
G0/G1
PI-A
PI-A
Supplementary Figure 5: Gating strategy for FACS
Gating strategy used for flow cytometry. The gate on the left was used to isolate live, single cells (used for analyses of both PI and EdU/PI), and the gate on the right was subsequently used to quantify EdU/PI profiles, based on the percentages of cells in G0/G1, S, and G2/M phases of the cell cycle.
9

## Slide 10
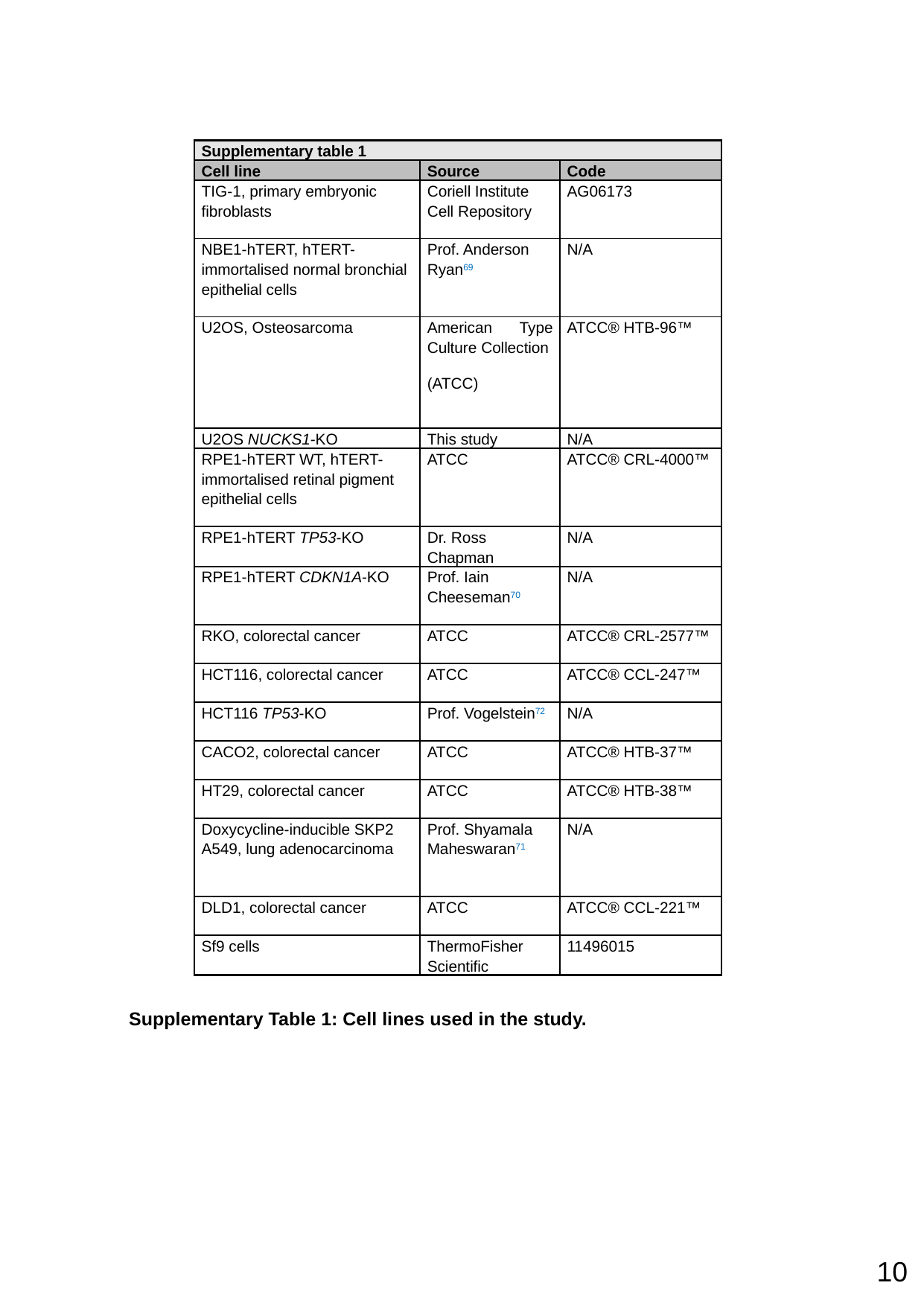

| Supplementary table 1 | | |
| --- | --- | --- |
| Cell line | Source | Code |
| TIG-1, primary embryonic fibroblasts | Coriell Institute Cell Repository | AG06173 |
| NBE1-hTERT, hTERT-immortalised normal bronchial epithelial cells | Prof. Anderson Ryan69 | N/A |
| U2OS, Osteosarcoma | American Type Culture Collection (ATCC) | ATCC® HTB-96™ |
| U2OS NUCKS1-KO | This study | N/A |
| RPE1-hTERT WT, hTERT-immortalised retinal pigment epithelial cells | ATCC | ATCC® CRL-4000™ |
| RPE1-hTERT TP53-KO | Dr. Ross Chapman | N/A |
| RPE1-hTERT CDKN1A-KO | Prof. Iain Cheeseman70 | N/A |
| RKO, colorectal cancer | ATCC | ATCC® CRL-2577™ |
| HCT116, colorectal cancer | ATCC | ATCC® CCL-247™ |
| HCT116 TP53-KO | Prof. Vogelstein72 | N/A |
| CACO2, colorectal cancer | ATCC | ATCC® HTB-37™ |
| HT29, colorectal cancer | ATCC | ATCC® HTB-38™ |
| Doxycycline-inducible SKP2 A549, lung adenocarcinoma | Prof. Shyamala Maheswaran71 | N/A |
| DLD1, colorectal cancer | ATCC | ATCC® CCL-221™ |
| Sf9 cells | ThermoFisher Scientific | 11496015 |
Supplementary Table 1: Cell lines used in the study.
10

## Slide 11
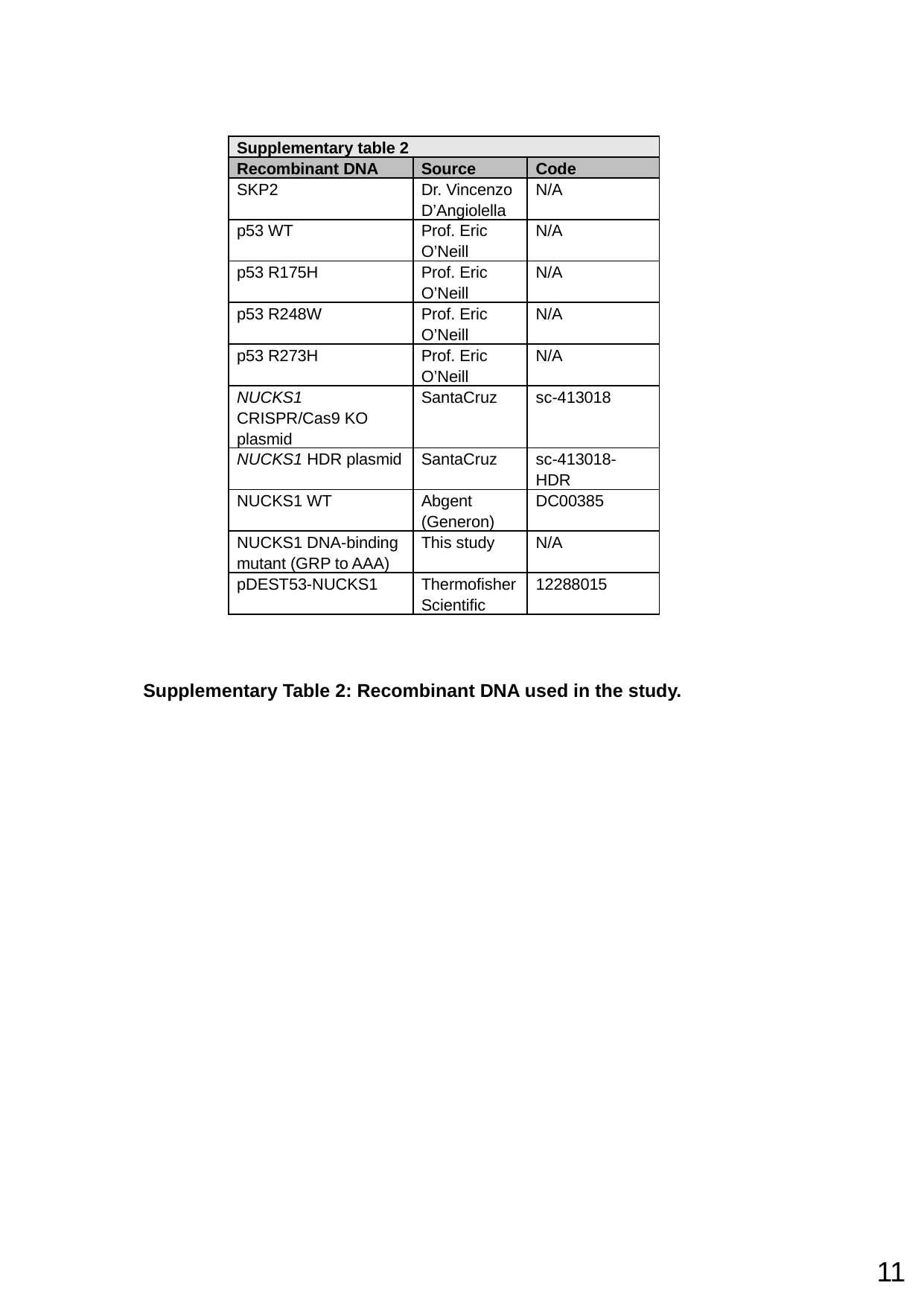

| Supplementary table 2 | | |
| --- | --- | --- |
| Recombinant DNA | Source | Code |
| SKP2 | Dr. Vincenzo D’Angiolella | N/A |
| p53 WT | Prof. Eric O’Neill | N/A |
| p53 R175H | Prof. Eric O’Neill | N/A |
| p53 R248W | Prof. Eric O’Neill | N/A |
| p53 R273H | Prof. Eric O’Neill | N/A |
| NUCKS1 CRISPR/Cas9 KO plasmid | SantaCruz | sc-413018 |
| NUCKS1 HDR plasmid | SantaCruz | sc-413018-HDR |
| NUCKS1 WT | Abgent (Generon) | DC00385 |
| NUCKS1 DNA-binding mutant (GRP to AAA) | This study | N/A |
| pDEST53-NUCKS1 | Thermofisher Scientific | 12288015 |
Supplementary Table 2: Recombinant DNA used in the study.
11

## Slide 12
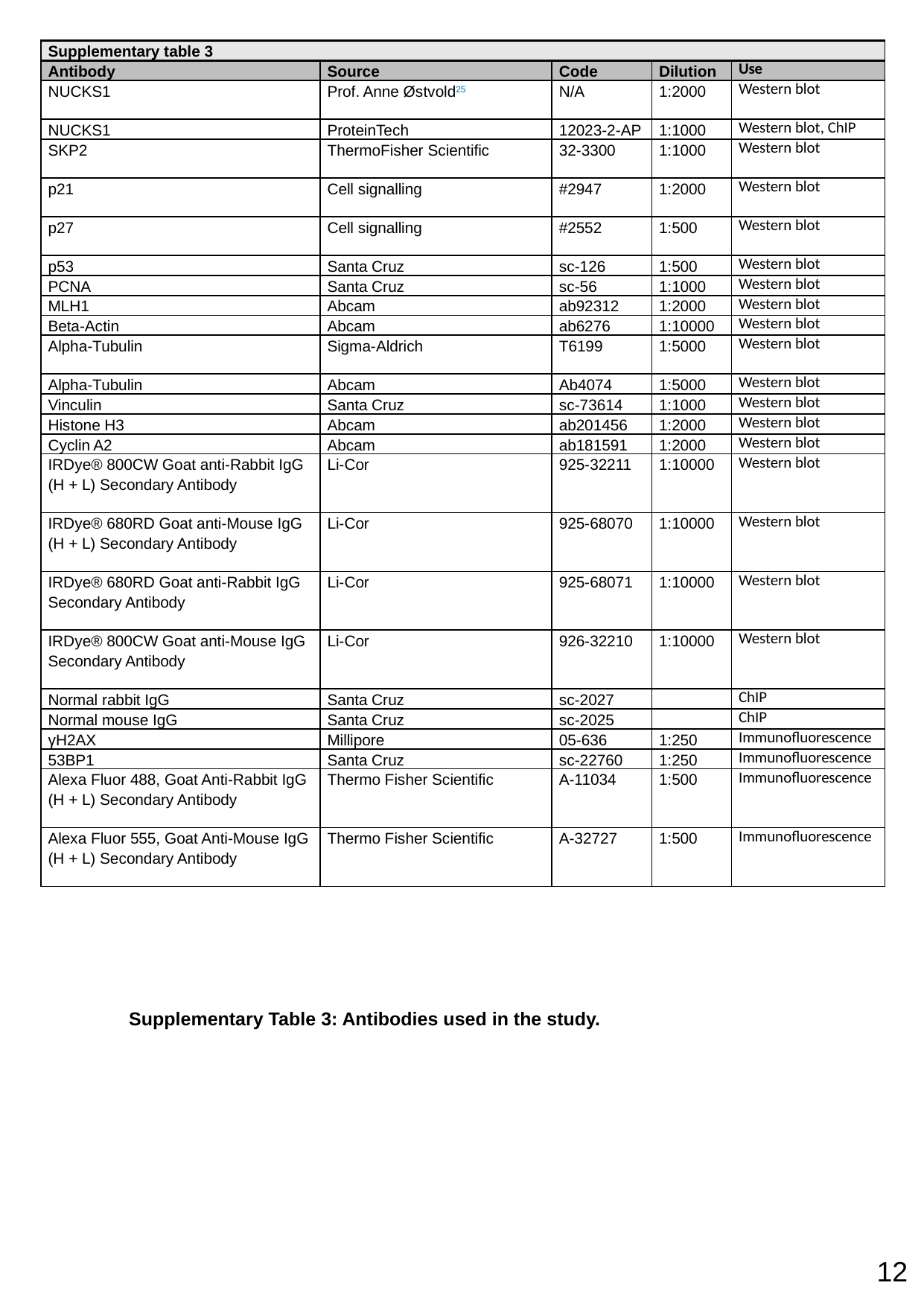

| Supplementary table 3 | | | | |
| --- | --- | --- | --- | --- |
| Antibody | Source | Code | Dilution | Use |
| NUCKS1 | Prof. Anne Østvold25 | N/A | 1:2000 | Western blot |
| NUCKS1 | ProteinTech | 12023-2-AP | 1:1000 | Western blot, ChIP |
| SKP2 | ThermoFisher Scientific | 32-3300 | 1:1000 | Western blot |
| p21 | Cell signalling | #2947 | 1:2000 | Western blot |
| p27 | Cell signalling | #2552 | 1:500 | Western blot |
| p53 | Santa Cruz | sc-126 | 1:500 | Western blot |
| PCNA | Santa Cruz | sc-56 | 1:1000 | Western blot |
| MLH1 | Abcam | ab92312 | 1:2000 | Western blot |
| Beta-Actin | Abcam | ab6276 | 1:10000 | Western blot |
| Alpha-Tubulin | Sigma-Aldrich | T6199 | 1:5000 | Western blot |
| Alpha-Tubulin | Abcam | Ab4074 | 1:5000 | Western blot |
| Vinculin | Santa Cruz | sc-73614 | 1:1000 | Western blot |
| Histone H3 | Abcam | ab201456 | 1:2000 | Western blot |
| Cyclin A2 | Abcam | ab181591 | 1:2000 | Western blot |
| IRDye® 800CW Goat anti-Rabbit IgG (H + L) Secondary Antibody | Li-Cor | 925-32211 | 1:10000 | Western blot |
| IRDye® 680RD Goat anti-Mouse IgG (H + L) Secondary Antibody | Li-Cor | 925-68070 | 1:10000 | Western blot |
| IRDye® 680RD Goat anti-Rabbit IgG Secondary Antibody | Li-Cor | 925-68071 | 1:10000 | Western blot |
| IRDye® 800CW Goat anti-Mouse IgG Secondary Antibody | Li-Cor | 926-32210 | 1:10000 | Western blot |
| Normal rabbit IgG | Santa Cruz | sc-2027 | | ChIP |
| Normal mouse IgG | Santa Cruz | sc-2025 | | ChIP |
| γH2AX | Millipore | 05-636 | 1:250 | Immunofluorescence |
| 53BP1 | Santa Cruz | sc-22760 | 1:250 | Immunofluorescence |
| Alexa Fluor 488, Goat Anti-Rabbit IgG (H + L) Secondary Antibody | Thermo Fisher Scientific | A-11034 | 1:500 | Immunofluorescence |
| Alexa Fluor 555, Goat Anti-Mouse IgG (H + L) Secondary Antibody | Thermo Fisher Scientific | A-32727 | 1:500 | Immunofluorescence |
Supplementary Table 3: Antibodies used in the study.
12

## Slide 13
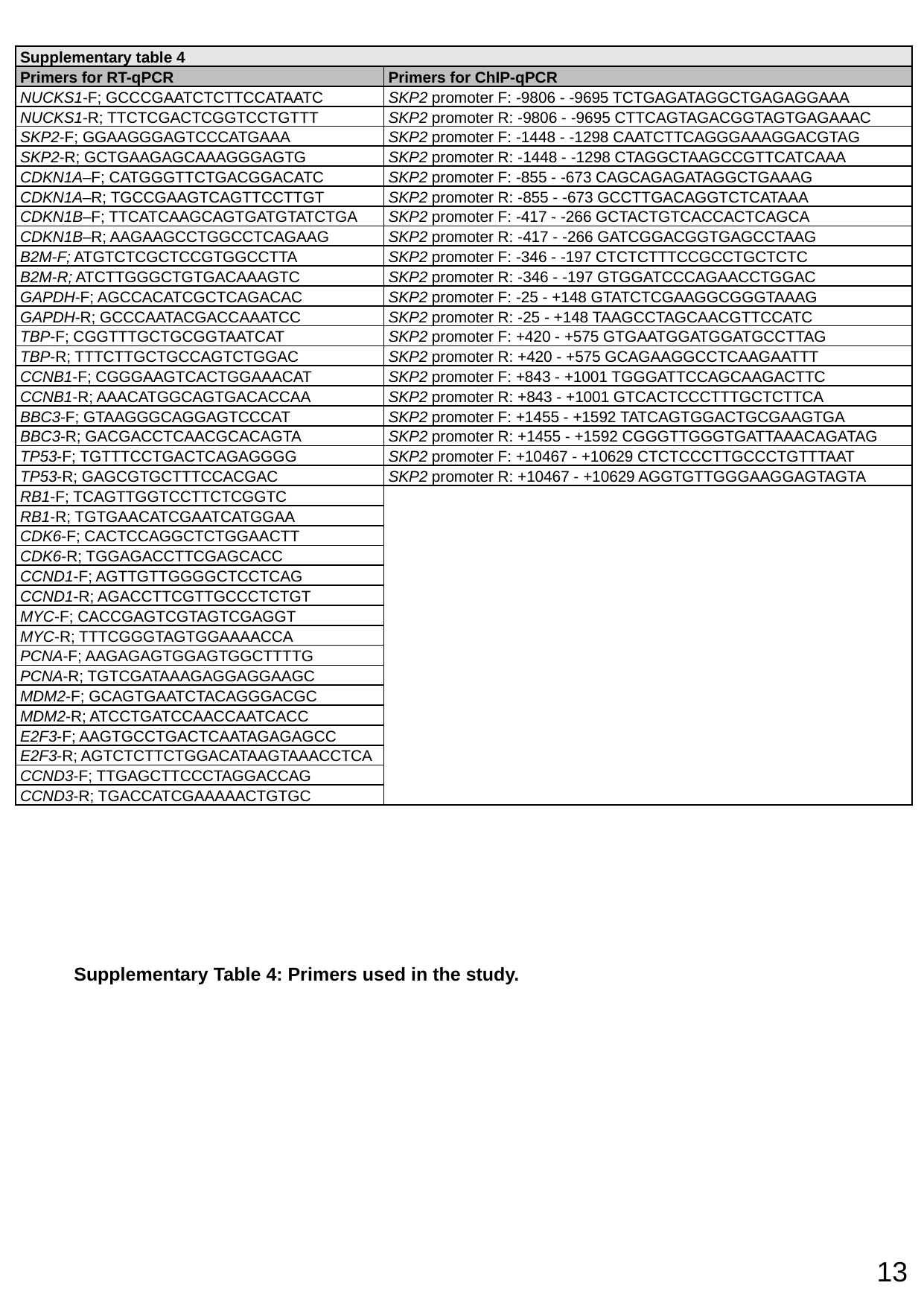

| Supplementary table 4 | |
| --- | --- |
| Primers for RT-qPCR | Primers for ChIP-qPCR |
| NUCKS1-F; GCCCGAATCTCTTCCATAATC | SKP2 promoter F: -9806 - -9695 TCTGAGATAGGCTGAGAGGAAA |
| NUCKS1-R; TTCTCGACTCGGTCCTGTTT | SKP2 promoter R: -9806 - -9695 CTTCAGTAGACGGTAGTGAGAAAC |
| SKP2-F; GGAAGGGAGTCCCATGAAA | SKP2 promoter F: -1448 - -1298 CAATCTTCAGGGAAAGGACGTAG |
| SKP2-R; GCTGAAGAGCAAAGGGAGTG | SKP2 promoter R: -1448 - -1298 CTAGGCTAAGCCGTTCATCAAA |
| CDKN1A–F; CATGGGTTCTGACGGACATC | SKP2 promoter F: -855 - -673 CAGCAGAGATAGGCTGAAAG |
| CDKN1A–R; TGCCGAAGTCAGTTCCTTGT | SKP2 promoter R: -855 - -673 GCCTTGACAGGTCTCATAAA |
| CDKN1B–F; TTCATCAAGCAGTGATGTATCTGA | SKP2 promoter F: -417 - -266 GCTACTGTCACCACTCAGCA |
| CDKN1B–R; AAGAAGCCTGGCCTCAGAAG | SKP2 promoter R: -417 - -266 GATCGGACGGTGAGCCTAAG |
| B2M-F; ATGTCTCGCTCCGTGGCCTTA | SKP2 promoter F: -346 - -197 CTCTCTTTCCGCCTGCTCTC |
| B2M-R; ATCTTGGGCTGTGACAAAGTC | SKP2 promoter R: -346 - -197 GTGGATCCCAGAACCTGGAC |
| GAPDH-F; AGCCACATCGCTCAGACAC | SKP2 promoter F: -25 - +148 GTATCTCGAAGGCGGGTAAAG |
| GAPDH-R; GCCCAATACGACCAAATCC | SKP2 promoter R: -25 - +148 TAAGCCTAGCAACGTTCCATC |
| TBP-F; CGGTTTGCTGCGGTAATCAT | SKP2 promoter F: +420 - +575 GTGAATGGATGGATGCCTTAG |
| TBP-R; TTTCTTGCTGCCAGTCTGGAC | SKP2 promoter R: +420 - +575 GCAGAAGGCCTCAAGAATTT |
| CCNB1-F; CGGGAAGTCACTGGAAACAT | SKP2 promoter F: +843 - +1001 TGGGATTCCAGCAAGACTTC |
| CCNB1-R; AAACATGGCAGTGACACCAA | SKP2 promoter R: +843 - +1001 GTCACTCCCTTTGCTCTTCA |
| BBC3-F; GTAAGGGCAGGAGTCCCAT | SKP2 promoter F: +1455 - +1592 TATCAGTGGACTGCGAAGTGA |
| BBC3-R; GACGACCTCAACGCACAGTA | SKP2 promoter R: +1455 - +1592 CGGGTTGGGTGATTAAACAGATAG |
| TP53-F; TGTTTCCTGACTCAGAGGGG | SKP2 promoter F: +10467 - +10629 CTCTCCCTTGCCCTGTTTAAT |
| TP53-R; GAGCGTGCTTTCCACGAC | SKP2 promoter R: +10467 - +10629 AGGTGTTGGGAAGGAGTAGTA |
| RB1-F; TCAGTTGGTCCTTCTCGGTC | |
| RB1-R; TGTGAACATCGAATCATGGAA | |
| CDK6-F; CACTCCAGGCTCTGGAACTT | |
| CDK6-R; TGGAGACCTTCGAGCACC | |
| CCND1-F; AGTTGTTGGGGCTCCTCAG | |
| CCND1-R; AGACCTTCGTTGCCCTCTGT | |
| MYC-F; CACCGAGTCGTAGTCGAGGT | |
| MYC-R; TTTCGGGTAGTGGAAAACCA | |
| PCNA-F; AAGAGAGTGGAGTGGCTTTTG | |
| PCNA-R; TGTCGATAAAGAGGAGGAAGC | |
| MDM2-F; GCAGTGAATCTACAGGGACGC | |
| MDM2-R; ATCCTGATCCAACCAATCACC | |
| E2F3-F; AAGTGCCTGACTCAATAGAGAGCC | |
| E2F3-R; AGTCTCTTCTGGACATAAGTAAACCTCA | |
| CCND3-F; TTGAGCTTCCCTAGGACCAG | |
| CCND3-R; TGACCATCGAAAAACTGTGC | |
Supplementary Table 4: Primers used in the study.
13
