# Supplementary material for: The NUCKS1-SKP2-p21/p27 axis controls S phase entry
Source: Nat Commun. 2021 Nov 29;12:6959. doi: 10.1038/s41467-021-27124-8 (PMC8630071; doi:10.1038/s41467-021-27124-8)
Supplement: Supplementary file 2 — Reporting Summary [file 41467_2021_27124_MOESM2_ESM.pdf]

nature portfolio

Corresponding author(s): Dr. Arnaud Legrand, Prof. Kristijan Ramadan, Prof. Grigory Dianov

Last updated by author(s): 22.9.21

Reporting Summary

Nature Portfolio wishes to improve the reproducibility of the work that we publish. This form provides structure for consistency and transparency in reporting. For further information on Nature Portfolio policies, see our Editorial Policies and the Editorial Policy Checklist.

Statistics

For all statistical analyses, confirm that the following items are present in the figure legend, table legend, main text, or Methods section.

n/a	Confirmed	
☐	☒	The exact sample size (*n*) for each experimental group/condition, given as a discrete number and unit of measurement
☐	☒	A statement on whether measurements were taken from distinct samples or whether the same sample was measured repeatedly
☐	☒	The statistical test(s) used AND whether they are one- or two-sided<br>*Only common tests should be described solely by name; describe more complex techniques in the Methods section.*
☒	☐	A description of all covariates tested
☐	☒	A description of any assumptions or corrections, such as tests of normality and adjustment for multiple comparisons
☐	☒	A full description of the statistical parameters including central tendency (e.g. means) or other basic estimates (e.g. regression coefficient) AND variation (e.g. standard deviation) or associated estimates of uncertainty (e.g. confidence intervals)
☐	☒	For null hypothesis testing, the test statistic (e.g. F, t, r) with confidence intervals, effect sizes, degrees of freedom and P value noted<br>*Give P values as exact values whenever suitable.*
☒	☐	For Bayesian analysis, information on the choice of priors and Markov chain Monte Carlo settings
☒	☐	For hierarchical and complex designs, identification of the appropriate level for tests and full reporting of outcomes
☐	☒	Estimates of effect sizes (e.g. Cohen's d, Pearson's r), indicating how they were calculated

Our web collection on statistics for biologists contains articles on many of the points above.

Software and code

Policy information about availability of computer code

Data collection	FACS Calibur, 7500 Fast Real-Time PCR System, FACS CytoFLEX, Odyssey image analysis system, Nikon NiE, Countess Automated Cell Counter
Data analysis	Graphpad Prism 8, FlowJo v10.6.1, Andor Komet7.1, Image Studio Lite Ver 5.2, ModFit LT 4.1.7, CellProfiler, 7500 Software v2.0.6.

For manuscripts utilizing custom algorithms or software that are central to the research but not yet described in published literature, software must be made available to editors and reviewers. We strongly encourage code deposition in a community repository (e.g. GitHub). See the Nature Portfolio guidelines for submitting code & software for further information.

Data

Policy information about availability of data

All manuscripts must include a data availability statement. This statement should provide the following information, where applicable:

- Accession codes, unique identifiers, or web links for publicly available datasets
- A description of any restrictions on data availability
- For clinical datasets or third party data, please ensure that the statement adheres to our policy

All source data are provided in the Source Data file.

Publicly-available datasets used:

SEEK (Zhu et al., Nature Methods 2015) http://seek.princeton.edu/
Genecodis3 (Tabas-Madrid et al., Nucleic Acids Research 2012) http://genecodis.cnb.csic.es
GEPIA2 (Tang et al., Nucleic Acids Research 2019) http://gepia2.cancer-pku.cn/#index

UCSC XENA (Goldman et al., Nature Biotechnology 2020) https://xena.ucsc.edu/
CBioPortal (Ceramin et al., Cancer Discovery 2012; Gao et al., Science Signaling 2013) https://www.cbioportal.org/

Field-specific reporting

Please select the one below that is the best fit for your research. If you are not sure, read the appropriate sections before making your selection.

- [x] Life sciences
- [ ] Behavioural & social sciences
- [ ] Ecological, evolutionary & environmental sciences

For a reference copy of the document with all sections, see nature.com/documents/nr-reporting-summary-flat.pdf

Life sciences study design

All studies must disclose on these points even when the disclosure is negative.

Sample size	For flow cytometry, at least 10,000 cells were measured per condition.<br>For immunofluorescence, over 100 cells were quantified per condition.<br>For comet assays, over 50 cells were quantified per test condition.<br>Most experiments were repeated two or three times, and most phenotypes were interrogated from multiple angles. For example, we measured SKP2 mRNA levels following loss of NUCKS1 in multiple cell lines, using different siRNAs as well as NUCKS1-knockout cells. This was further confirmed at the protein level, using multiple cell lines.<br>Most of our bioinformatics analyses include the results of hundreds of patients.<br><br>These sample sizes were chosen based on the current standards in the literature.
Data exclusions	No relevant data were excluded from the study.
Replication	Most experiments were repeated 2-3 times, and all replication attempts were successful. Most phenotypes were further investigated using orthogonal approaches to increase reliability.
Randomization	Different cell samples started from similar conditions and treatments were then randomly allocated.
Blinding	Blinding was not performed. Many experiments (for example Western blots) rely on knowledge of sample identity to load in a logical order.

Reporting for specific materials, systems and methods

We require information from authors about some types of materials, experimental systems and methods used in many studies. Here, indicate whether each material, system or method listed is relevant to your study. If you are not sure if a list item applies to your research, read the appropriate section before selecting a response.

Materials & experimental systems

n/a	Involved in the study	
☐	☒	Antibodies
☐	☒	Eukaryotic cell lines
☒	☐	Palaeontology and archaeology
☒	☐	Animals and other organisms
☒	☐	Human research participants
☒	☐	Clinical data
☒	☐	Dual use research of concern

Methods

n/a	Involved in the study	
☒	☐	ChIP-seq
☐	☒	Flow cytometry
☒	☐	MRI-based neuroimaging

Antibodies

Antibodies used	NUCKS1 (kind gift from Anne Østvold), NUCKS1 (ProteinTech 12023-2-AP), SKP2 (Thermofisher 32-3300), p21 (Cell signaling #2947), p27 (Cell signaling #2552), p53 (Santa Cruz sc-126), PCNA (Santa Cruz sc-56), MLH1 (Abcam ab92312), Beta-Actin (Abcam ab6276), Alpha-Tubulin (Sigma T6199), Alpha-Tubulin (Abcam ab4074), Vinculin (Santa Cruz sc-73614), Histone H3 (Abcam ab201456), Cyclin A2 (Abcam ab181591), IRDye® 800CW Goat anti-Rabbit IgG (H + L) (Li-Cor 925-32211), IRDye® 680RD Goat anti-Mouse IgG (H + L) (Li-Cor 925-68070), IRDye® 680RD Goat anti-Rabbit IgG Secondary Antibody (Li-Cor 925-68071), IRDye® 800CW Goat anti-Mouse IgG Secondary Antibody (Li-Cor 926-32210), Normal rabbit IgG (Santa Cruz sc-2027), Normal mouse IgG (Santa Cruz sc-2025), γH2AX (Millipore 05-636), 53BP1 (Santa Cruz sc-22760), Alexa Fluor 488, Goat Anti-Rabbit IgG (H + L) (Thermofisher A-11034), Alexa Fluor 555, Goat Anti-Mouse IgG (H + L) (Thermofisher A-32727).
Validation	Antibodies were validated by siRNA-mediated depletion, because they react as expected in response to treatment (for example the cyclin A2 antibody detects a protein only expressed in S/G2 phase, the 53BP1 antibody forms foci in response to DNA damage, the

p53 antibody is upregulated in response to DNA damage), by molecular weight using Western blot, and/or by the manufacturers:

NUCKS1 12023-2-AP: validated by manufacturer for Western blot and IP
SKP2 32-3300: validated by manufacturer for Western blot
p21 2947: validated by manufacturer for Western blot
p27 2552: validated by manufacturer for Western blot
p53 sc-126: validated by manufacturer for Western blot
PCNA sc-56: validated by manufacturer for Western blot
MLH1 ab92312: validated by manufacturer for Western blot
Actin ab6276: validated by manufacturer for Western blot
Tubulin T6199: validated by manufacturer for Western blot
Tubulin ab4074: validated by manufacturer for Western blot
Vinculin sc-73614: validated by manufacturer for Western blot
Histone H3 ab201456: validated by manufacturer for Western blot
Cyclin A2 ab181591: validated by manufacturer for Western blot
γH2AX 05-636: validated by manufacturer for immunofluorescence
53BP1 sc-22760: validated by manufacturer for immunofluorescence

Eukaryotic cell lines

Policy information about cell lines

Cell line source(s)	TIG-1 (Coriell Institute Cell Repository AG06173), NBE1-hTERT (Prof. Anderson Ryan), U2OS (ATCC® HTB-96™), U2OS NUCKS1-KO (this study), RPE1-hTERT (ATCC® CRL-4000™), RPE1-hTERT TP53-KO (Dr. Ross Chapman), RPE1-hTERT CDKN1A-KO (Prof. Iain Cheeseman), RKO (ATCC® CRL-2577™), HCT116 (ATCC® CCL-247™), HCT116 TP53-KO (Prof. Vogelstein), CACO2 (ATCC® HTB-37™), HT29 (ATCC® HTB-38™), Doxycycline-inducible SKP2 A549 (Prof. Shyamala Maheswaran), DLD1 (ATCC® CCL-221™).
Authentication	Cell lines were not authenticated by us.
Mycoplasma contamination	All cell lines tested negative for mycoplasma, using the MycoAlertTM Mycoplasma Detection Kit (Lonza LR07-218).
Commonly misidentified lines (See ICLAC register)	No commonly misidentified cell lines were used in the study.

Flow Cytometry

Plots

Confirm that:

- [x] The axis labels state the marker and fluorochrome used (e.g. CD4-FITC).
- [x] The axis scales are clearly visible. Include numbers along axes only for bottom left plot of group (a 'group' is an analysis of identical markers).
- [x] All plots are contour plots with outliers or pseudocolor plots.
- [x] A numerical value for number of cells or percentage (with statistics) is provided.

Methodology

Sample preparation	For propidium iodide staining, trypsinised cells were fixed in cold 70% ethanol for 30 min on ice. Cells were then centrifuged at 250 g for 5 min and resuspended in PBS with 0.5 µg/ml RNAseA and 10 µg/ml propidium iodide, before incubation for 15 min at 37 °C.<br>For EdU/PI staining, the Click-iT™ Plus EdU Alexa Fluor™ 488 Flow Cytometry Assay Kit was used, according to the manufacturer's instructions.
Instrument	The FACSCalibur and CytoFLEX machines were used.
Software	FlowJo v10.6.1, ModFit LT 4.1.7
Cell population abundance	The percentage of cells in G0/G1, S and G2/M phases was assessed.
Gating strategy	Single, live cells were isolated using a plot of prodidium iodide vs. width (Supplementary Figure 5).

- [x] Tick this box to confirm that a figure exemplifying the gating strategy is provided in the Supplementary Information.
